# ﻿Five new species of *Trichoderma* from moist soils in China

**DOI:** 10.3897/mycokeys.87.76085

**Published:** 2022-02-17

**Authors:** Guang-Zhi Zhang, He-Tong Yang, Xin-Jian Zhang, Fang-Yuan Zhou, Xiao-Qing Wu, Xue-Ying Xie, Xiao-Yan Zhao, Hong-Zi Zhou

**Affiliations:** 1 Qilu University of Technology (Shandong Academy of Sciences), Ecology Institute, Shandong Provincial Key Laboratory for Applied Microbiology, Jinan 250103, China Qilu University of Technology Jinan China

**Keywords:** Hypocreales, phylogenetic analysis, soil fungi, Sordariomycetes, taxonomy

## Abstract

*Trichoderma* isolates were collected from moist soils near a water source in different areas of China. ITS sequences were submitted to MIST (Multiloci Identification System for *Trichoderma*) and meets the *Trichoderma* [ITS_76_] standard. Combined analyses of phylogenetic analyses of both phylograms (*tef1*-α and *rpb2*) and morphological characteristics, revealed five new species of *Trichoderma*, namely *Trichodermahailarense*, *T.macrofasciculatum*, *T.nordicum*, *T.shangrilaense* and *T.vadicola*. Phylogenetic analyses showed *T.macrofasciculatum* and *T.shangrilaense* belong to the Polysporum clade, *T.hailarense*, while *T.nordicum* and *T.vadicola* belong to the Viride clade. Each new taxon formed a distinct clade in phylogenetic analysis and have unique sequences of *tef1*-α and *rpb2* that meet the *Trichoderma* new species standard. The conidiation of *T.macrofasciculatum* typically appeared in white pustules in concentric rings on PDA or MEA and its conidia had one or few distinctly verrucose. Conidiophores of *T.shangrilaense* are short and rarely branched, phialides usually curved and irregularly disposed. The aerial mycelium of *T.hailarense* and *T.vadicola* formed strands to floccose mat, conidiation tardy and scattered in tufts, conidiophores repeatedly rebranching in dendriform structure. The phialides of *T.nordicum* lageniform are curved on PDA and its conidia are globose to obovoidal and large.

## ﻿Introduction

The genus *Trichoderma* belongs to one of the most useful groups of microbes to have had an impact on human welfare in recent times. They are most widely used as biofungicides and plant growth modifiers and are sources of enzymes of industrial utility, including those used in the biofuels industry (Mukherjee et al. 2013). Some *Trichoderma* species have great potential applications to remediate soil and water pollution (Tripathi et al. 2013). *Trichoderma* is a hyperdiverse fungal genus (Jaklitsch and Voglmayr 2015). Formerly the species-level identification of *Trichoderma* was performed, based on their morphological characteristics (Gams and Bissett 1998) and is becoming more and more difficult because there are only a few relatively invariable morphological characteristics, leading to overlap amongst species (Samuels 2006).

DNA sequence analysis was introduced and provided more reliable identification of *Trichoderma* species (Druzhinina et al. 2006; Samuels 2006; Samuels et al. 2006). Given their low sequence variability or missing adequate sequence data, ITS, *cal1* and *chi18-5* are rarely used for new *Trichoderma* species identifications (Bissett et al. 2015; Cai and Druzhinina 2021). *Tef1*-α and *rpb2* facilitate reliable species identifications through phylogenetic analyses (Bissett et al. 2015; Jaklitsch and Voglmayr 2015; Cai and Druzhinina 2021) and have been used in the phylogenetic analysis and identification of new *Trichoderma* species in recent years. This has resulted in the exponential expansion of *Trichoderma* taxonomy, with up to 20 new species recognised per year (Cai and Druzhinina 2021). As of July 2021, a total of 405 species has been reported and recognised (Bustamante et al. 2021; Cai and Druzhinina 2021; Rodríguez et al. 2021; Zheng et al. 2021). The new molecular identification protocol provides a standard for the molecular identification of *Trichoderma* (Cai and Druzhinina 2021; www.trichoderma.info). According to this protocol, the new species should meet the *Trichoderma* [ITS_76_] standard and has unique sequences of *rpb2* or *tef1* (does not meet the *sp*∃!(*rpb2*_99_≅*tef1*_97_) standard for known species).

*Trichoderma* species are cosmopolitan and prevalent components of different ecosystems in a wide range of climatic zones (Kubicek et al. 2008). They are mainly found in natural soils and decaying wood and plant material (Kredics et al. 2014). Many new *Trichoderma* species were first discovered in China, with up to 115 new *Trichoderma* species being reported since 2016 (Zhu and Zhuang 2015a, b, 2018; Chen and Zhuang 2016, 2017a, b, c, d; Qin and Zhuang 2016a, b, c, 2017; Sun et al. 2016; Zeng and Zhuang 2017, 2019; Zhang and Zhuang 2017, 2018, 2019; Li et al. 2018; Qiao et al. 2018; Zhao et al. 2018; Ding et al. 2020; Gu et al. 2020; Liu et al. 2020; Zheng et al. 2021). Amongst these 115 species, 75 were isolated from soils, 36 were collected from the plant branch or rotten twigs, while the other four species were collected from mushroom, pollen or rotten fruit.

*Trichoderma* has been segregated into many clades (Bissett 1991; Atanasova et al. 2013). The Polysporum clade (formerly section Pachybasium) was first defined by Bissett (1991), including 20 species. However, molecular phylogeny has shown that it is paraphyletic (Kindermann et al. 1998; Kullnig-Gradinger et al. 2002) and the species composition was subdivided subsequently into five unrelated clades, such as Ceramica, Harzianum, Semiorbis, Strictipilosa or Stromaticum (Chaverri and Samuels 2003; Jaklitsch 2009; Jaklitsch 2011). *Trichodermahamatum* and some other species were found to belong to the section Trichoderma. The removal of *T.hamatum* determined that Bissett’s sectional name could not be used anymore. Lu et al. (2004) refined the clade containing the remaining species around *T.polysporum/Hypocrea pachybasioides* and it was named the *Pachybasium* core group by Jaklitsch (2011), which includes 13 species. In subsequent years, several new species were added to this clade, increasing the number of *Trichoderma* species to 21 species (Jaklitsch and Voglmayr 2015; Zhu and Zhuang 2015; Qin and Zhuang 2016c; Chen and Zhuang 2017b).

The Virde clade is basically in accordance with Bissett’s (1991) concept, but later, some other species have been added constantly. As of 2015, this large clade has 72 species to be confirmed and described, amongst which 55 species have been well located in the six subclades (Hamatum/Asperellum, Koningii, Neorufum, Rogersonii, Viride and Viridescens) and 17 species have not been located in the unnamed branches (Park et al. 2014; Bissett et al. 2015; Jaklitsch and Voglmayr 2015). In subsequent years, 25 new species were added to this clade, increasing the number of *Trichoderma* species to 97 (Montoya et al. 2016; Qin and Zhuang 2016a; Chen and Zhuang 2017c; Zeng et al. 2017; Zhang and Zhuang 2017; du Plessis et al. 2018; Qiao et al. 2018; Zhang and Zhuang 2018; Crous et al. 2019; Ding et al. 2020; Tomah et al. 2020). Cai and Druzhinina (2021) reconstructed the core topology of the phylogram, based on the Maximum Likelihood (ML) phylogeny of the 361 *rpb2*-barcoded *Trichoderma* species and 361 species have been located in the eight main clades (numerically named 1–8). All *Trichoderma* species in the adjacent Polysporum and Viride clades were remerged into the 5^th^ clade, which also included several *Trichoderma* species from the *Harzianum* and *lone lineage* clades (Jaklitsch and Voglmayr 2015; Sun et al. 2016; Zhang and Zhuang 2017).

The present study performed the phylogenetic analysis of the five new species of *Trichoderma* to establish their new status. Five new species were collected from moist soils near water in different areas of China. *Tef1*-α and *rpb2* sequences were used for the phylogenetic reconstruction of the five new species in the present study and meet the *Trichoderma* new species standard (Cai and Druzhinina 2021).

## ﻿Materials and methods

### ﻿Isolates and specimens

Specimens were collected from Sichuan, Yunnan, Beijing, Shandong and Inner Mongolia. *Trichoderma* strains were isolated from soils on *Trichoderma* Selective Medium (K_2_HPO_4_ 0.90 g; MgSO_4_·7H_2_O 0.20 g; NH_4_NO_3_ 1.0 g; KCl 0.15 g; glucose 3.0 g; Rose Bengal 0.15 g; Agar 15.0 g; distilled water 1.0 litre. Post autoclaving, chloromycetin (0.25 g), streptomycin (0.03 g) and pentachloronitrobenzene (0.2 g) were added) (Martin 1950). Ex-type living cultures of new species were deposited in the Agricultural Culture Collection of China (ACCC) (Institute of Agricultural Resources and Regional Planning, Chinese Academy of Agricultural Sciences, Beijing, China).

### ﻿Morphological characterisations

Morphological observation of the colonies and conidium-bearing structures was based on isolates grown on PDA (potato dextrose agar, Difco), CMD (Difco cornmeal agar + 2% w/v dextrose), MEA (malt extract agar, Difco) and Nirenberg’s SNA medium (Nirenberg 1976) for 2 weeks in an incubator at 25 °C with alternating 12 h/12 h fluorescent light/darkness. Microscopic observations were conducted with an Olympus BX53 microscope and a MicroPublisher 5.0 RTV digital camera (Olympus Corp., Tokyo, Japan). Continuous characters, such as length and width, were measured with the CellSens Standard Image software (Olympus Corp., Tokyo, Japan). Continuous measurements were based on 10–30 measured units and were reported as the extremes (maximum and minimum) in brackets separated by the mean plus and minus one standard deviation. Colour standards were from Kornerup and Wanscher (1978). Growth-rate trials were performed on 9 cm Petri dishes with 20 ml of CMD, PDA, MEA and SNA at 15 °C, 20 °C, 25 °C, 30 °C, and 35 °C. Petri dishes were incubated in darkness up to 1 week or until the colony covered the agar surface. Colony radii were measured daily. Trials were replicated three times.

### ﻿DNA extraction, polymerase chain reaction (PCR) and sequencing

Strains were grown in 9 cm-diameter Petri dishes containing PDA (potato dextrose agar, Difco). Cultures were incubated at 25 °C for ca. 3–5 days. Genomic DNA was extracted from the mycelial mat harvested from the surface of the broth with the Fungal Genomic DNA Extraction Kit (Aidlab Biotechnologies Co. Ltd., Beijing, China). The amplification of ITS was performed using the primer pair ITS5 and ITS4 (White et al. 1990), for *tef1*-α, primer pair EF1-728F (Carbone and Kohn 1999) and tef1-ΑLLErev (Jaklitsch et al. 2005) was used and, for *rpb2*, primer pair frpb2-5f and frpb2-7cr (Liu et al. 1999) was used. PCR amplification of each gene was performed as described by Park et al. (2014) and Chaverri et al. (2011). PCR products were purified and sequenced by ABI3730 Gene Analyzer at Sangon (Sangon Biotech (Shanghai) Co., Ltd.).

### ﻿Molecular identification and phylogenetic analyses

We followed the molecular identification protocol for a single *Trichoderma* isolate (Cai and Druzhinina 2021; www.trichoderma.info) and estimated the pairwise similarity between the ITS sequence of the query strain and the sequences that are given in the ITS56 datasets (Cai and Druzhinina 2021). *Tef1*-α and *rpb2* sequences were subjected to Multiloci Identification System for *Trichoderma* (MMIT) (mmit.china-cctc.org) (Dou et al. 2020) and NCBI nucleotide BLAST (https://blast.ncbi.nlm.nih.gov/Blast.cgi) to detect the most closely related species. A sufficient number of representative sequences (n > 6) of *Trichoderma* species (Bissett et al. 2015; Cai and Druzhinina 2021) that are closely related to the new species were chosen for phylogenetic analyses. *Protocreaillinoensis* and *Protocreafarinose* were selected as outgroups.

Sequences were aligned with ClustalW (Thompson et al. 1994) and adjusted manually. Gaps were treated as missing data. Phylogenetic analyses were performed with *tef1*-α or *rpb2* with MEGA-X software (Kumar et al. 2018). Model testing was used to find the best DNA model for ML analyses. The stability of clades was evaluated by bootstrap tests with 1000 replications. Bootstrap values above 50% were indicated on the corresponding branches. Maximum Parsimony (MP) analyses were performed with MEGA-X software (Kumar et al. 2018) using 1000 replicates of heuristic search with the random addition of sequences and tree bisection reconnection as the MP search method. All molecular characters were weighted equally and gaps were treated as missing data. Bootstrap proportions were calculated from 1000 replicates, each with 10 replicates of random addition of taxa.

## ﻿Results

### ﻿Molecular identification and sequence analyses

We estimated the pairwise similarity between the ITS sequence of the query strain and the sequences that are given in the ITS56 datasets. All the query strain belongs to the genus *Trichoderma* spp. with similarity value > 81% compared to the sequences in the datasets. The query strain has unique sequences of *tef1*-α and *rpb2* (does not meet the *sp*∃!(*rpb2*_99_≅*tef1*_97_) standard for known *Trichoderma* species).

**Figure 1.**
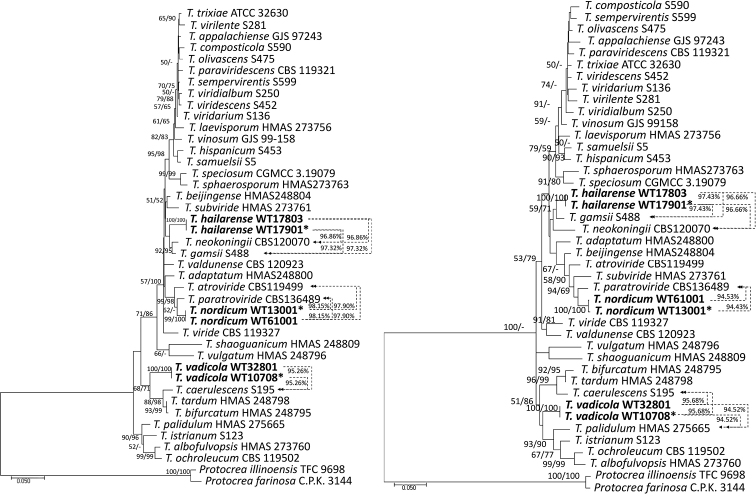
Phylogenetic tree, based on the Maximum Likelihood analysis of the *rpb2* (left; InL = -5930.92) and *tef1*-α (right; InL = -7681.95) dataset. Bootstrap values of Maximum Likelihood (left) and Maximum Parsimony (right) above 50% are indicated at the nodes. The tree is rooted with *Protocreaillinoensis* TFC 9698 and *P.farinose* CPK 3144. New species proposed here are indicated in bold. The type strains are indicated with an asterisk (*) after the strain number. Results of the pairwise sequence similarity are illustrated on the dashed lines between the query strain and its closely-related species (arrows point to the reference strains).

**Figure 2.**
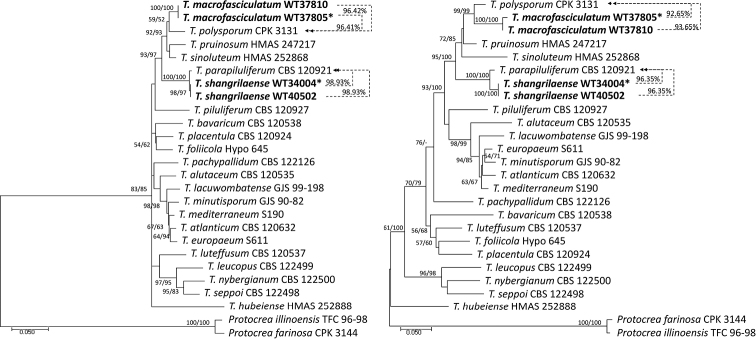
Phylogenetic tree based on the Maximum Likelihood analysis of the *rpb2* (left; InL = -5912.02) and *tef1*-α (right; InL = -9060.53) dataset. Maximum Likelihood bootstrap values (left) and MPBP (right) above 50% are indicated at the nodes. The tree is rooted with *Protocreaillinoensis* TFC 9698 and *P.farinose* CPK 3144. New species proposed here are indicated in bold. The type strains are indicated with an asterisk (*) after the strain number. Results of the pairwise sequence similarity are illustrated on the dashed lines between the query strain and its closely-related species (arrows point to the reference strains).

**Figure 3.**
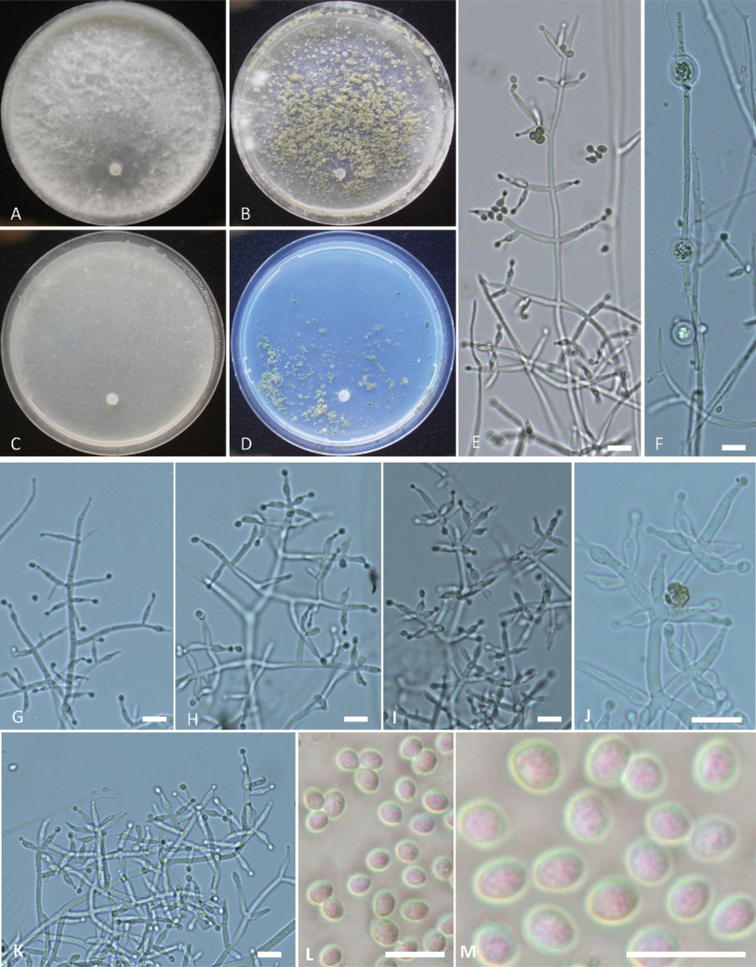
*Trichodermahailarense***A–D** cultures on different media incubated at 25 °C for 14 days (**A** on PDA**B** on MEA**C** on CMD**D** on SNA) **E, G–K** conidiophores and phialides **F** chlamydospores **L, M** conidia. Notes: **E** on MEA**F–M** on PDA**A–M** from WT17901. Scale bars: 10 μm (**E–J**).

**Figure 4.**
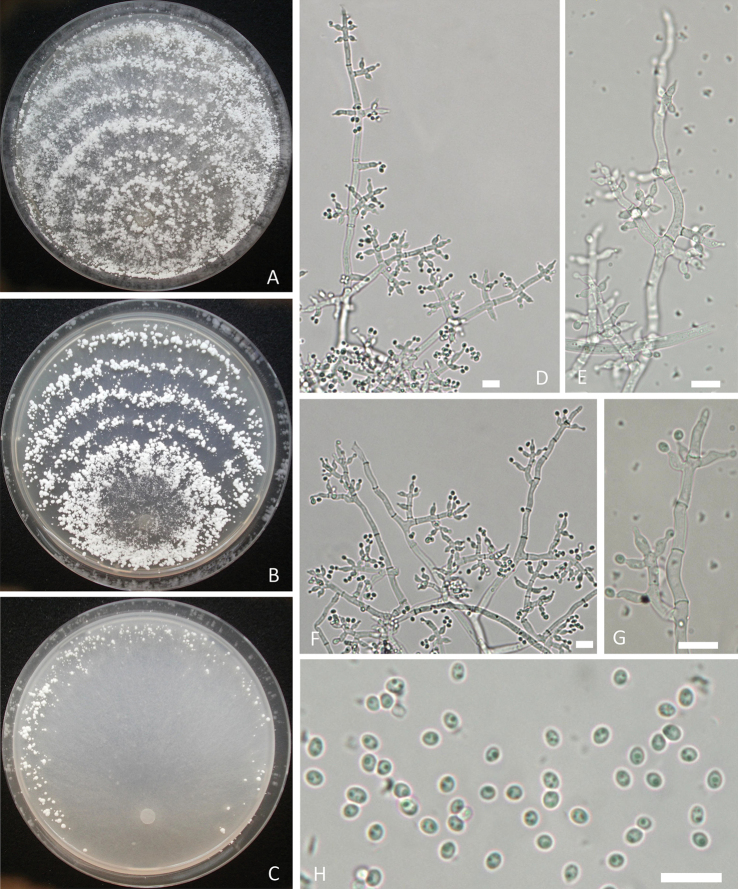
*Trichodermamacrofasciculatum***A–C** cultures on different media incubated at 25 °C for 7 days (**A** on PDA**B** on MEA**C** on CM) **D–G** conidiophores and phialides **H** conidia with guttules. Notes: **A, D, E** from WT37810 **B, C, F, G** from WT37805. Scale bars: 10 μm (**D–H**).

*Tef1*-α or *rpb2* sequences of new taxon were subjected to MMIT and NCBI nucleotide BLAST and 34 representative sequences of *Trichoderma* species (all the species with similarity *rpb2* and *tef1*-α ≥ 92% in the Viride clade) that are closely related to the new species, were chosen for phylogenetic analyses of *T.hailarense*, *T.nordicum* and *T.vadicola*. The accession numbers for the sequences are provided in Table 1. Model testing suggested using the Hasegawa-Kishino-Yano model (HKY; Hasegawa 1985) with gamma distributed with invariant sites (HKY+G+I) for ML analyses of *tef1*-α and the Tamura-Nei model (TN93; Tamura 1993) with gamma distributed substitution rates (TN93+G) for *rpb2*. The phylogenetic trees from *rpb2* or *tef1*-α analyses are shown in Figs 1 and 2, respectively. Sequence alignments and the trees obtained were deposited in TreeBASE (http://purl.org/phylo/treebase/phylows/study/TB2:S29166). Twenty representative sequences of closely-related *Trichoderma* species (all the *Trichoderma* species in the Polysporum clade) were chosen for phylogenetic analyses of *T.macrofasciculatum* and *T.shangrilaense* (Table 1). Model testing suggested using the Hasegawa-Kishino-Yano model (HKY; Hasegawa 1985) with gamma distributed substitution rates (HKY+G) for ML analyses of *tef1*-α and the Kimura 2-parameter (K2; Kimura 1980) with gamma distributed substitution rates (K2+G) for *rpb2*. The phylogenetic trees from *rpb2* or *tef1*-α analyses are shown in Figs 3 and 4, respectively. Sequence alignments and the trees obtained were deposited in TreeBASE (http://purl.org/phylo/treebase/phylows/study/TB2:S29166).

**Table 1.** Strain numbers and GenBank accession numbers of sequences used for phylogenetic analyses.

Species	Clade	Strain	GenBank accession numbers		
			ITS	*tef1*-α	*rpb2*
*T.adaptatum*	Viride	HMAS 248800	–	KX428024	KX428042
*T.albofulvopsis*	Viride	HMAS 273760	–	KU529127	KU529138
*T.alutaceum*	Polysporum	CBS 120535	FJ860725	FJ179567	FJ179600
*T.appalachiense*	Viride	GJS 97-243	DQ315419	DQ307503	DQ307503
*T.atlanticum*	Polysporum	CBS 120632	FJ860781	FJ860649	FJ860546
*T.atroviride*	Viride	CBS 119499	FJ860726	FJ860611	FJ860518
*T.bavaricum*	Polysporum	CBS 120538	FJ860737	FJ860621	FJ860527
*T.beijingense*	Viride	HMAS 248804	–	KX428025	KX428043
*T.bifurcatum*	Viride	HMAS 248795	–	KX428018	KX428036
*T.caerulescens*	Viride	S195	JN715589	JN715621	JN715604
*T.composticola*	Viride	S590=CBS 133497	–	KC285631	KC285754
*T.europaeum*	Polysporum	S611	–	KJ665489	KJ665268
*T.foliicola*	Polysporum	Hypo 645	JQ685871	JQ685862	JQ685876
*T.gamsii*	Viride	S488	–	JN715613	KJ665270
***T.hailarense***	**Viride**	WT17901*= ACCC 39711	**MH287485**	**MH287505**	**MH287506**
***T.hailarense***	**Viride**	**WT17803**	**MH606226**	**MH606229**	**MH606232**
*T.hispanicum*	Viride	S453=CBS 130540	JN715595	JN715659	JN715600
*T.istrianum*	Viride	S123	–	KJ665521	KJ665280
*T.laevisporum*	Viride	HMAS 273756	–	KU529128	KU529139
*T.lacuwombatense*	Polysporum	GJS 99-198	–	KJ665547	KJ665286
*T.leucopus*	Polysporum	CBS 122499	FJ860764	FJ179571	FJ179605
*T.luteffusum*	Polysporum	CBS 120537	FJ860773	FJ860645	FJ860543
***T.macrofasciculatum***	**Polysporum**	WT37805* = ACCC 39712	**MH287487**	**MH287509**	**MH287493**
***T.macrofasciculatum***	**Polysporum**	**WT37810**	**MH287488**	**MH287510**	**MH287494**
*T.mediterraneum*	Polysporum	S190	–	KJ665568	KJ665296
*T.minutisporum*	Polysporum	GJS 90-82	–	KJ665618	KJ665316
*T.neokoningii*	Viride	CBS 120070=GJS 04-216	DQ841734	KJ665620	KJ665318
***T.nordicum***	**Viride**	WT13001* =ACCC 39713	**MH287483**	**MH287501**	**MH287502**
***T.nordicum***	**Viride**	**WT61001**	**MH287484**	**MH287503**	**MH287504**
*T.nybergianum*	Polysporum	CBS 122500	FJ860791	FJ179575	FJ179611
*T.ochroleucum*	Viride	CBS 119502	FJ860793	FJ860659	FJ860556
*T.olivascens*	Viride	S475=CBS 132574	–	KC285624	KC285752
*T.pachypallidum*	Polysporum	CBS 122126	FJ860798	FJ860662	JQ685879
*T.palidulum*	Viride	HMAS 275665	–	MG383493	MG383487
*T.paratroviride*	Viride	CBS136489	–	KJ665627	KJ665321
*T.paraviridescens*	Viride	CBS 119321	DQ677651	DQ672610	KC285763
*T.parapiluliferum*	Polysporum	CBS 120921	FJ860799	FJ179578	FJ179614
*T.piluliferum*	Polysporum	CBS 120927	FJ860810	FJ860674	FJ179615
*T.placentula*	Polysporum	CBS 120924	–	FJ179580	FJ179616
*T.polysporum*	Polysporum	CPK 3131	–	FJ860661	FJ860558
*T.pruinosum*	Polysporum	HMAS 247217	–	MF371227	MF371212
*T.samuelsii*	Viride	S5=CBS 130537	JN715593	JN715651	JN715599
*T.sempervirentis*	Viride	S599=CBS 133498	–	KC285632	KC285755
*T.seppoi*	Polysporum	CBS 122498	–	FJ179581	FJ179617
***T.shangrilaense***	**Polysporum**	WT34004*= ACCC 39714	**MH287489**	**MH287495**	**MH287496**
***T.shangrilaense***	**Polysporum**	**WT40502**	**MH606224**	**MH606227**	**MH606230**
*T.shaoguanicum*	Viride	HMAS 248809	–	KX428031	KX428049
*T.sinoluteum*	Polysporum	HMAS 252868	–	KJ634777	KJ634744
*T.speciosum*	Viride	CGMCC 3.19079	MH113929	MH183184	MH155270
*T.sphaerosporum*	Viride	HMAS 273763	–	KU529134	KU529145
*T.subviride*	Viride	HMAS 273761	–	KU529131	KU529142
*T.tardum*	Viride	HMAS 248798	–	KX428020	KX428038
*T.trixiae*	Viride	ATCC 32630	DQ315445	DQ307526	KC285770
***T.vadicola***	**Viride**	WT10708*= ACCC 39716	**MH287491**	**MH287499**	**MH287511**
***T.vadicola***	**Viride**	**WT32801**	**MH606225**	**MH606228**	**MH606231**
*T.valdunense*	Viride	CBS 120923	FJ860863	FJ860717	FJ860605
*T.vinosum*	Viride	GJS 99-158=CBS 119087	AY380904	AY376047	KC285779
*T.viridarium*	Viride	S136=CBS 132568	–	KC285658	KC285760
*T.viride*	Viride	CBS 119327	DQ677655	DQ672617	EU711362
*T.viridescens*	Viride	S452=CBS 132573	–	KC285646	KC285758
*T.viridialbum*	Viride	S250=CBS 133495	–	KC285706	KC285774
*T.virilente*	Viride	S281=CBS 132569	–	KC285692	KC285767
*T.vulgatum*	Viride	HMAS 248796	–	KX428019	KX428037
*Protocreaillinoensis*	Outgroup	TFC 96-98	EU703930	EU703905	EU703952
*Protocreafarinosa*	Outgroup	CPK 3144	EU703917	EU703894	EU703938

Newly-sequenced material is indicated in bold type.

The MP analyses using *tef1*-α and *rpb2* (Fig. 1) resulted in topologically similar trees with minor differences. Each new taxon of *Trichoderma* formed a distinct clade and meets the *Trichoderma* new species standard (does not meet the *sp*∃!(*rpb2*_99_≅*tef1*_97_) standard for known *Trichoderma* species) (Cai and Druzhinina 2021). The similarity value between the new species and the reference strain is shown in the number on the right side of the phylogenetic trees.

*Trichodermahailarense* clearly separated from *T.gamsii* S488 (with similarity *rpb2* = 97.32% and *tef1*-α = 97.43%) and *T.neokoningii* CBS120070 (with similarity *rpb2* = 96.86% and *tef1*-α = 96.66%). *Trichodermanordicum* was associated, but clearly separated from *T.paratroviride* CBS136489 with similarity *rpb2* = 98.15% and *tef1* = 94.43%. *Trichodermavadicola* was associated, but clearly separated from *T.caerulescens* S195 (with similarity 95.26%), *T.tardum* HMAS 248798 (with similarity 95.57%) and *T.bifurcatum* S195 (with similarity 95.76%) in the phylogenetic tree of the *rpb2*. However, there were differences in the phylogenetic tree of the *tef1*-α; *T.vadicola* was associated and separated from *T.palidulum* HMAS 275665 (with similarity 94.52%), *T.istrianum* S123 (with similarity 96.14%), *T.ochroleucum* CBS 119502 (with similarity 93.49%) and *T.albofulvopsis* HMAS 273760 (with similarity 93.16%) (Fig. 1). The strains of *T.macrofasciculatum* were associated, but clearly separated from *T.polysporum* C.P.K. 3131 with similarity *rpb2* = 96.41% and *tef1*-α = 92.81%; *T.shangrilaense* was closely related and separated from *T.parapiluliferum* CBS 120927 with similarity *rpb2* = 98.93% and *tef1*-α = 96.35% (Fig. 2).

### ﻿Taxonomy

#### ﻿ Trichoderma hailarense

Taxon classificationFungiHypocrealesHypocreaceae

G.Z. Zhang sp. nov.

9E2BEFC1-73D8-56DC-A035-342FB5D4251A

821318

Fig. 3

##### Etymology.

The specific epithet “*hailarense*” refers to the locality, the Hailar River Basin in Inner Mongolia of China where the holotype was found.

##### Typification.

China. Inner Mongolia, Hailar River Basin, 618 m (altitude), isolated from soil, 17 September 2016, G.Z. *Zhang* (Holotype WT 17901).

##### Diagnosis.

Phylogenetically, *Trichodermahailarense* formed a distinct clade and is related to *T.gamsii* and *T.neokoningii* (Fig. 1). The sequence similarity of *rpb2* with *T.gamsii* S488 and *T.neokoningii* CBS120070 was 97.32% and 96.86%, respectively and the sequence similarity of *tef1*-α with *T.gamsii* S488 and *T.neokoningii* CBS120070 was 97.43% and 96.66%, respectively. Colonies of *T.hailarense* did not form conidia on PDA and conidia of *T.hailarense* on other media were obovoid, delicately roughened and easily distinguished from those of *T.gamsii* and *T.neokoningii*.

##### Teleomorph.

Unknown.

Growth optimal at 30 °C, slow at 35 °C on all media. Colony radius after 72 h at 30 °C 53–56 mm on PDA, 54–56 mm on CMD, 33–37 mm on MEA and 33–36 mm on SNA. Colony radius after 72 h at 35 °C 13–15 mm on PDA, 10–14 mm on CMD, 9–12 mm on MEA and 10–12 mm on SNA. Aerial mycelia abundant, arachnoid on PDA after 72 h at 25 °C under 12 h photoperiod. Conidiation started around the inoculation point after 7 days on PDA, with relatively few or small conidia. Diffusing pigment or distinctive odour absent. Conidiation started around the inoculation point after 7 days on MEA, forming a few large pustules, cream yellow. On SNA, aerial mycelia were few, forming a few large pustules around the inoculation point in age, cream-yellow. Conidiophores and branches narrow and flexuous, tending to be regularly verticillate, forming a pyramidal structure, with each branch terminating in a cruciate whorl of up to five phialides. Phialides, lageniform, (8.0–)9.4–13.1(–15.5) × (2.5–)3.0–3.5(–3.6) μm (mean = 11.2 × 3.3 μm), base 1.8–2.5 μm (mean = 2.1 μm); phialide length/width ratio (2.33–)2.7–4.4(–5.9) (mean = 3.4). Conidia obovoid, (4.2–)4.3–4.7(–4.9) × (3.4–)3.6–3.9(–4.1) μm (mean = 4.5 × 3.7 μm), length/width ratio 1.1–1.4 (mean = 1.2), delicately roughened. Chlamydospores: (7.0–)7.5–8.2(–8.5) × (6.5–)7.0–7.5(–8.3) μm.

##### Distribution.

China. Inner Mongolia.

##### Additional specimen examined.

China. Inner Mongolia, Hulun Buir, 610 m (altitude), isolated from soil, 17 September 2016, *J.D. Hu* (WT17905).

##### Notes.

Phylogenetically *Trichodermahailarense* is related to *T.gamsii* and *T.neokoningii* (Fig. 1) and does not meet the *sp*∃!(*rpb2*_99_≅*tef1*_97_) standard for *T.gamsii* or *T.neokoningii*. Morphologically, colonies of *T.gamsii* and *T.neokoningii* on PDA formed conidia sporadically or in hemispherical pustules and conidia of *T.gamsii* and *T.neokoningii* were ellipsoidal to oblong, smooth-walled (Jaklitsch et al. 2006). However, colonies of *T.hailarense* did not form conidia on PDA and conidia of *T.hailarense* on other media were obovoid, delicately roughened and easily distinguished from those of *T.gamsii* and *T.neokoningii*.

#### ﻿ Trichoderma macrofasciculatum

Taxon classificationFungiHypocrealesHypocreaceae

G.Z. Zhang sp. nov.

78B37AB8-8A21-52F5-B563-06B4AA0094BE

821299

Fig. 4

##### Etymology.

The specific epithet “*macrofasciculatum*” refers to the morphological feature of the conidiation, conidiophores aggregated into large fascicles in concentric rings.

##### Typification.

China, Sichuan, Nine-Village Valley, 2405 m (altitude), isolated from soil, 24 September 2016, G.Z. *Zhang* (Holotype WT 37805).

##### Diagnosis.

Phylogenetically, *Trichodermamacrofasciculatum* WT37805 and WT37810 formed a distinct clade and is related to *T.polysporum* C.P.K. 3131 in the Polysporum clade, but the similarities of *rpb2* and *tef1*-α between these two species were only 96.41% and 92.81%, respectively. *Trichodermamacrofasciculatum* cannot grow at 35 °C as *T.polysporum* and the former formed large and white pustules in concentric rings at 25 °C, elongations were rarely observed and conidia had few guttules, which are distinct from *T.polysporum*.

##### Teleomorph.

Unknown.

Growth optimum at 20 °C, slow or limited at 30 °C, absent at 35 °C. Colony radius after 72 h at 25 °C 21–24 mm on PDA, 23–27 mm on CMD, 17–20 mm on MEA and 12–16 mm on SNA. Aerial mycelia abundant on PDA and MEA after incubation for 72 h at 25 °C under a 12 h photoperiod. Conidiation typically in pustules in concentric rings on PDA, solitary or aggregated, producing a farinose to granular mat. Diameter of pustules up to 2.2 mm, pompon-like, white. Diffusing pigment and distinct odour absent. Conidiation on MEA typically in pustules in concentric rings, pompon-like as on PDA. On CMD, aerial mycelia sparsely developed. Conidiation aggregated in sporadic pustules near the colony margin, white. On SNA, aerial mycelia few and conidiation not observed. Conidiophores and branches irregularly branched in a dendriform structure, with each branch terminating in a cruciate whorl of up to five phialides. Hyphal septa clearly visible. Phialides flask-shaped, often curved, (4.9–)5.6–7.8(–8.8) × (2.8–)3.0–3.2(–3.4) μm (mean = 6.7 × 3.1 μm), 1.8–2.6 μm (mean = 2.2 μm) near the base; phialide length/width ratio (1.5–)1.8–2.4(–2.8) (mean = 2.1). Conidia subglobose to ellipsoid, hyaline, smooth, with one or few distinctly verrucose, (2.6–)2.8–3.3(–3.6) × (2.4–)2.5–2.7(–2.9) μm (mean = 3.0 × 2.6 μm), length/width ratio 1.0–1.3 (mean = 1.2). Chlamydospores not observed.

##### Distribution.

China, Sichuan Province.

##### Additional material examined.

China, Sichuan, Nine-Village Valley, 2405 m (altitude), isolated from soil, 24 September 2016, G.Z. *Zhang* (WT 37810).

##### Notes.

Phylogenetically *Trichodermamacrofasciculatum* WT 37805 is related to *T.polysporum* C.P.K. 3131 in the Polysporum clade (Fig. 1), but the similarities of *rpb2* and *tef1*-α between these two species were only 96.41% and 92.81% respectively, with 94 and 41 bp differences amongst 1311 and 1152 bp. *Trichodermamacrofasciculatum* cannot grow at 35 °C as *T.polysporum* and the former formed large and white pustules in concentric rings at 25 °C, elongations were rarely observed and conidia had few guttules, which are distinct from *T.polysporum* (Lu et al. 2004).

#### ﻿ Trichoderma nordicum

Taxon classificationFungiHypocrealesHypocreaceae

G.Z. Zhang sp. nov.

DE284B67-F384-59E0-92E8-05DCBFD3BCE4

8212301

Fig. 5

##### Etymology.

“nord” means found in the north of China.

##### Holotype.

China, Beijing, Yu-yuan-tan Park, 43 m (altitude), isolated from soil, 27 October 2016, G.Z. Zhang (Holotype WT 13001), ex-type culture ACCC 39713.

##### Diagnosis.

Phylogenetically *Trichodermanordicum* is related to *T.paratroviride*, but the sequence similarities of *rpb2* and *tef1*-α were 98.15% and 94.43%, respectively. That does not meet the *sp*∃!(*rpb2*_99_≅*tef1*_97_) standard for *T.paratroviride* or other known *Trichoderma* species. Morphologically, conidiophores of *T.paratroviride* consisting of a main axis and often distantly-spaced side branches, not re-branching. Conidiophores of *T.nordicum* are branched in a more complex manner; conidia are larger than those of *T.paratroviride*.

##### Teleomorph.

Unknown.

Growth optimal at 25 °C, slow or limited at 30 °C, absent at 35 °C. Colonies grew fast on PDA, CMD and MEA and slow on SNA. Colony radius after 72 h at 25 °C 67–71 mm on PDA, 68–71 mm on CMD, 51–55 mm on MEA and 21–24 mm on SNA. Aerial mycelia sparse on PDA after 72 h at 25 °C under 12 h photoperiod and conidiation developed within 48 h beginning at the inoculation point and progressed around, grey-white at first and slowly turning green. Diffusing pigment or distinctive odour absent. Aerial mycelia sparse and flocculence on MEA after 72 h at 20 °C under 12 h photoperiod. Conidia developed within 48 h beginning near the colony margin on MEA, grey-white at first and slowly turning green, transparent liquid secreted. Aerial mycelia few on SNA and CMD after 72 h at 25 °C, conidia formed around the inoculation point and in distinct concentric rings after 96 h under 12 h photoperiod on SNA and CMD, diffusing pigment not produced. Conidiophores and branches narrow and flexuous, tending to be regularly verticillate forming a pyramidal structure, each branch terminating in a cruciate whorl of up to five phialides. Phialides, lageniform, (6.2–)7.2–10.3(–12.9) × (2.6–)2.9–3.2(–3.4) μm (mean = 8.8 × 3.1 μm), 1.6–2.3 μm (mean = 1.9 μm) near the base; phialide length/width ratio (2.1–)2.4–3.4(–4.3) (mean = 2.9). On PDA, phialides curved, distinguished from those on other media. Conidia, globose to obovoidal, (4.1–)4.4–4.8(–5.0) × (4.0–)4.1–4.4(–4.6) μm (mean = 4.6 × 4.3 μm), length/width ratio 1.0–1.2 (mean = 1.1). Chlamydospores sometimes present, (8.7–)9.8 × 10.4(–12.5) μm.

**Figure 5.**
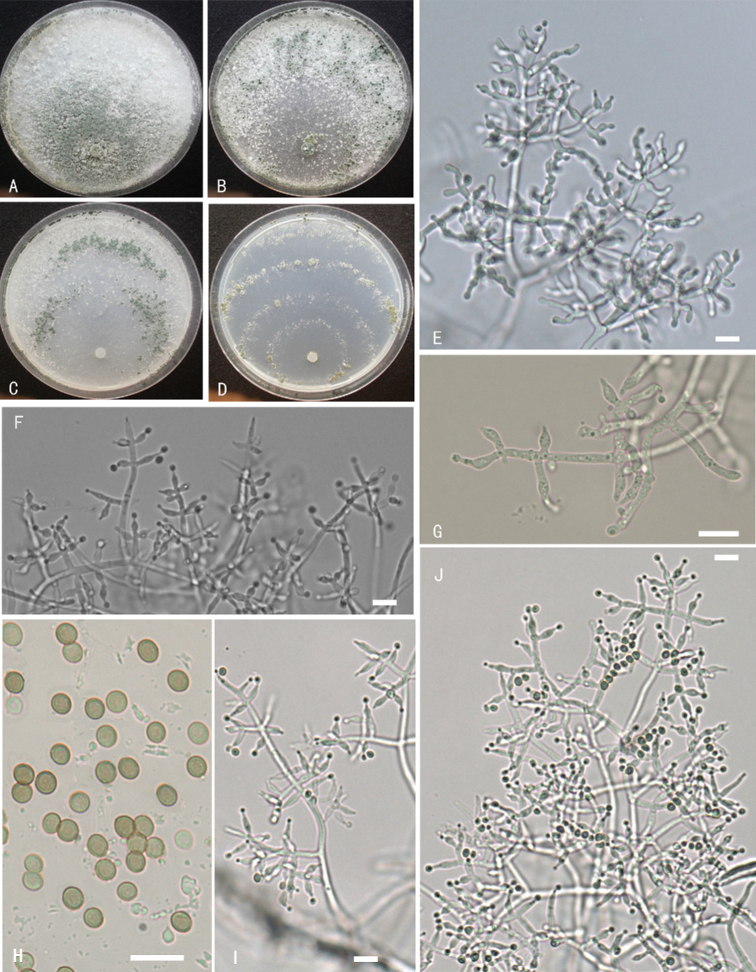
*Trichodermanordicum***A–D** cultures on different media at 25 °C after 10 days (**A** on PDA**B** on MEA**C** on CMD**D** on SNA) **E–G, I, J** conidiophores and phialides **H** conidia. Notes: **E** on PDA**F–J** on MEA**A–D** from WT13001 **E–J** from WT61001. Scale bars: 10 μm (**E–J**).

##### Distribution.

China, Beijing and Hebei.

##### Additional specimen examined.

China. Hebei, Bai-yang Lake, 19 m (altitude), isolated from soil, 15 September 2016, *J.S. Li* (WT 61001).

##### Notes.

Phylogenetically, *Trichodermanordicum* is related to *T.paratroviride* (Fig. 1), but the sequence similarities of *rpb2* and *tef1*-α were 98.15% and 94.43%, respectively. That does not meet the *sp*∃!(*rpb2*_99_≅*tef1*_97_) standard for *T.paratroviride* or other known *Trichoderma* species. Morphologically, conidiophores of *T.paratroviride* consist of a main axis and often distantly-spaced side branches, not re-branching. Conidiophores of *T.nordicum* are branched in a more complex manner; conidia are larger than those of *T.paratroviride* (Jaklitsch and Voglmayr 2015).

#### ﻿ Trichoderma shangrilaense

Taxon classificationFungiHypocrealesHypocreaceae

G.Z. Zhang sp. nov.

51ECA484-6FB6-5DD3-88CF-FFE20CD94506

821300

Fig. 6

##### Etymology.

“*shangrilaense*” was originally found at Shangrila in Yunnan Province of China.

##### Typification.

China. Yunnan, Pudacuo National Park, 3611 m (altitude), isolated from soil, 21 June 2016, G.Z. Zhang (Holotype WT 34004), Ex-type culture ACCC 39714.

##### Diagnosis.

Phylogenetically, *Trichodermashangrilaense* is related to *T.parapiluliferum* (CBS 120921) (Fig. 1), but the sequence similarity of *rpb2* between these two species was 98.93% and the sequence similarity of *tef1*-α was 96.35%. That does not meet the *sp*∃!(*rpb2*_99_≅*tef1*_97_) standard for *T.parapiluliferum* or other known *Trichoderma* species. Conidiophore main axis of *T.shangrilaense* fertile to apex, conidia obovoid to ellipsoid, easily distinguished from that of *T.parapiluliferum*.

##### Teleomorph.

Unknown.

Growth optimal at 20 °C, slow, limited at 25 °C and absent at 30 °C or 35 °C. Colony radius after 72 h at 20 °C 19–21 mm on PDA, 23–24 mm on CMD, 19–21 mm on MEA and 8–11 mm on SNA. Aerial mycelia abundant, compact on PDA after 7 days at 20 °C under 12 h photoperiod, conidiation not easily formed and a yellow diffusing pigment developed near the inoculation point; conidiation formed unequal in size, white pustules after 14 days. Conidiophores and branches narrow and flexuous, forming a dendriform structure and irregularly branched, not rebranched, main axis to 4.3–5.0 µm wide, fertile to apex. Phialides, flask-shaped, often curved, (4.5–)5.7–9.0(–11.1) × (2.9–)3.2–3.5(–4.1) μm (mean = 7.4 × 3.4 μm), 1.6–3.4 μm wide (mean = 2.6 μm) near the base; phialide length/width ratio (1.5–)2.0–2.6(–3.0) (mean = 2.3). Conidia, obovoid to ellipsoidal, smooth, (3.3–)3.5–4.0(–4.4) × (2.8–)3.0–3.3(–3.5) μm (mean = 3.8 × 3.19 μm), length/width ratio 1.1–1.4 (mean = 1.2). Chlamydospores not observed.

**Figure 6.**
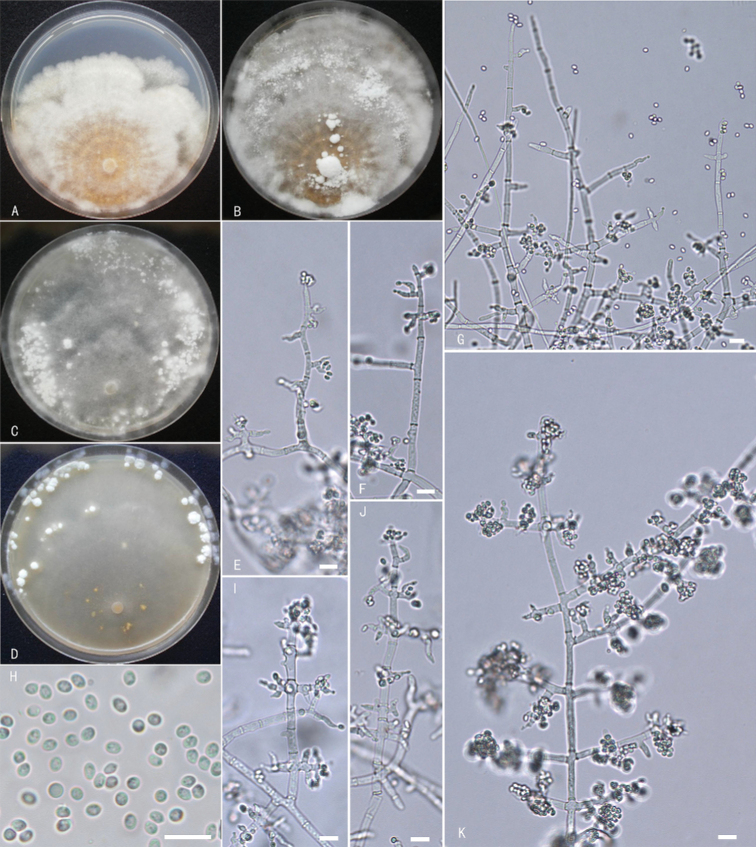
*Trichodermashangrilaense***A–D** cultures (**A** on PDA, 25 °C, 10 days **B** on PDA, 25 °C, 21 days **C** on MEA, 25 °C, 21 days **D** on CMD, 25 °C, 21 days) **E–G, I–K** conidiophores and phialides **H** conidia **A–K** from WT34004. Scale bars: 10 μm (**E–K**).

Colony radius 28–33 mm, aerial mycelia abundant and floccose after 7 days at 20 °C under 12 h photoperiod. Conidiation slowly developing on MEA. After about 14 days, pompon-like, white fascicles developed. No diffusing pigment observed. On CMD after 7 days at 20 °C under 12 h photoperiod, colony radius 28–33 mm, aerial mycelia few. Conidiation formed flat or cushion-shaped pustules near the colony margin after 21 days and a yellow diffusing pigment developed near the inoculation point. On SNA after 7 days at 20 °C under 12 h photoperiod, colony mycelia sparse and no conidiation formed. After 10 days, pustules scattered around the periphery of the colony. Diffusing pigment not developed.

##### Distribution.

China. Yunnan and Sichuan.

##### Additional specimen examined.

China. Sichuan, Huanglong Nature Reserve, 3561 m (altitude), isolated from soil, 25 September 2016, *Z. Li* (WT 34012).

##### Notes.

Phylogenetically, *Trichodermashangrilaense* is related to *T.parapiluliferum* (CBS 120921) (Fig. 1), but the sequence similarity of *rpb2* between these two species was 98.93% and the sequence similarity of *tef1*-α was 96.35%. The sequence similarity of *tef1*-α with the ex-type culture G.J.S. 91-60 (GenBank accession no. AY937444) was only 92%. Optimum temperature for growth of *T.shangrilaense* was 20 °C, no growth occurred at 30 °C as in *T.parapiluliferum* and conidiation structures consist of flat or cushion-shaped pustules, formed near the colony margin on MEA, SNA and CMD. Conidiophore main axis of *Trichodermaparapiluliferum* has conspicuous spiral sterile apical elongations, conidia ellipsoidal to oblong (Lu et al. 2004). Conidiophore main axis of *T.shangrilaense* fertile to apex, conidia obovoid to ellipsoid, easily distinguished from that of *T.parapiluliferum*.

#### ﻿ Trichoderma vadicola

Taxon classificationFungiHypocrealesHypocreaceae

G.Z. Zhang sp. nov.

929D624B-E67C-5359-9083-1E7A60EF07CB

821316

Fig. 7

##### Etymology.

The specific epithet “*vadicola*”, from the noun “vadum”, reflects the ecological environment and means that the species inhabits shallow water.

##### Typification.

China. Shandong, 2 m (altitude), isolated from soil, 13 August 2016, G.Z. Zhang (Holotype WT 10708), Ex-type culture ACCC 39716.

##### Diagnosis.

Phylogenetically, *Trichodermavadicola* is related to *T.caerulescens* in the Viride clade (Fig. 1), but the sequence similarity of *tef1*-α and *rpb2* between these species was all 95%. Morphologically, colonies of *T.vadicola* and *T.caerulescens* on PDA have similar features, such as abundant aerial hyphae, forming strands and a whitish hairy or floccose mat. However, the former *Trichodermavadicola* formed no or relatively few conidia and the latter forming greyish-bluish patches around the plug. On CMD, *T.caerulescens* peculiar greyish-blue pigment formed after 1–2 months and conidiophores simply or slightly branched; the former had no observed diffusing pigment and conidiophores branched in a complex manner in pyramidal structure or tree-like.

##### Teleomorph.

Unknown.

Growth optimal at 25 °C, no grow at 35 °C on all media. Colony radius after 72 h at 25 °C 25–29 mm on PDA, 24–27 mm on CMD, 23–26 mm on MEA and 22–26 mm on SNA. Aerial mycelia abundant on PDA after 72 h at 25 °C under 12 h photoperiod, forming strands and floccose mat. Conidiation not formed or relatively few. No diffusing pigment or distinctive odour was produced. On MEA after 72 h at 25 °C under 12 h photoperiod, aerial mycelia abundant, floccose. After 7 days, mycelia covered the plate and conidia appeared, effuse, granuliform. On CMD after 72 h at 25 °C under 12 h photoperiod, aerial mycelia not observed. After 7 days, mycelia covered the plate and conidia developed near the colony margin. On SNA after 72 h at 25 °C under 12 h photoperiod, aerial mycelia not observed. After 7 days, mycelia covered the plate, aerial mycelia floccose and conidia formed, effuse. Conidiophores and branches regularly verticillate, formed a pyramidal structure, each branch terminating in a cruciate whorl of 3–5 phialides. Phialides lageniform, (8.3–)9.9–12.3(–15.1) × (2.0–)2.6–3.2(–3.4) μm (mean = 11.1 × 2.9 μm), 1.1–2.9 μm wide (mean = 1.9 μm) near the base; phialide length/width ratio (2.7–)3.2–4.6(–6.6) (mean = 3.9). Conidia subglobose or obovoidal, (3.5–)3.7–4.3(–4.8) × (3.2–)3.4–3.6(–3.8) μm (mean = 4.0 × 3.5 μm), length/width ratio 1.0–1.3 (mean = 1.1). Chlamydospores not observed.

**Figure 7.**
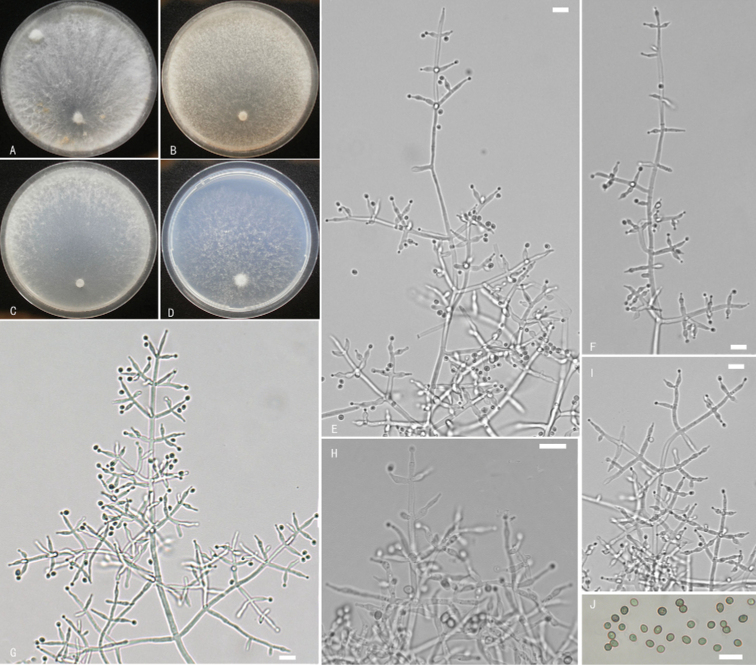
*Trichodermavadicola***A–D** cultures on different media at 25 °C (**A** on PDA after10 days **B** on MEA, after 7 days **C** on CMD after 7 days **D** on SNA after 7 days) **E–I** conidiophores and phialides **J** conidia. Notes: **E, F, H–J** on MEA**G** on SNA **A–J** from WT10708. Scale bars: 10 μm (**E–J**).

##### Distribution.

China. Shandong and Yunnan Provinces.

##### Additional specimen examined.

China. Yunnan, Shangri-La, Pudacuo National Park, 3551 m (altitude), isolated from soil, 21 September 2016, H.T. Yang (WT 10713).

##### Notes.

Phylogenetically, *Trichodermavadicola* is related to *T.caerulescens* in the Viride clade (Fig. 1), but the sequence similarity of *tef1*-α and *rpb2* between these species was all 95%, with 62 and 60 bp differences amongst 1218 and 1130 bp, respectively. Morphologically, colonies of *T.vadicola* and *T.caerulescens* on PDA have similar features, such as abundant aerial hyphae, forming strands and a whitish hairy or floccose mat. However, the former *Trichodermavadicola* formed no or relatively few conidia, with the latter forming greyish-bluish patches around the plug. On CMD, *T.caerulescens* formed peculiar greyish-blue pigment after 1–2 months and conidiophores simply or slightly branched (Jaklitsch et al. 2012); the former had no observed diffusing pigment and conidiophores branched in a complex manner in pyramidal structure or tree-like.

## ﻿Discussion

In this paper, five new species of *Trichoderma* were described from wetland soils. An ML tree was reconstructed, based on individual *tef1*-α and *rpb2*, to explore the taxonomic positions of the new species. Our phylogenetic analyses showed that the five new *Trichoderma* species belong to the Polysporum clade or the Virde clade. *Trichodermamacrofasciculatum* and *T.shangrilaense* belong to the Polysporum clade (as *Pachybasium* core group; Jaklitsch 2011) (Fig. 2). Here, we added two new species, *T.macrofasciculatum* and *T.shangrilaense*, which are close to *T.polysporum* and *T.parapiluliferum*. Morphologically, species in this clade are heterogeneous, comprising teleomorphs with upright, stipitate or small pulvinate stromata. The teleomorphs of *T.macrofasciculatum* and *T.shangrilaense* have not been found at present, but their asexual characteristics, such as conidiation in white pustules, resemble other species in this clade.

*Trichodermanordicum*, *T.vadicola*, and *T.hailarense* belong to the Viride clade (formerly section Trichoderma) (Fig. 1). Here, we added three new species, *T.hailarenseT.nordicum* and *T.vadicola*, which are all located in the unnamed branches and close to *T.gamsii*/*T.neokoningii*, *T.paratroviride* and *T.caerulescens*, respectively. Phenotypically, phialides of three new species are lageniform and have green conidia, which is consistent with the characteristics of *Trichoderma* species in the Viride clade. Only *T.hailarense* has coarsely warted conidia, two other species being smooth-walled.

At present, the identification of *Trichoderma* species is mainly based on phylogenetic analysis and morphological characteristics. The new species hypothesis needs to be supported by the topology of both phylograms (*rpb2* and *tef1*-α). However, there are no numerical standards of the similarity threshold at the level which is sufficient for identification for most of the existing species (Cai and Druzhinina 2021) and this has led to many inaccuracies in the original identification of *Trichoderma*. In the phylogenetic tree constructed in this paper, some *Trichoderma* species combinations showed low bootstrap values (Figs 1 and 2) and have high similarity, which meet the *sp*∃!(*rpb2*_99_≅*tef1*_97_) standard developed by Cai and Druzhinina (2021). They may be identified as the same *Trichoderma* species: for example, *T.viridialbum*, *T.viridarium* and *T.sempervirentis*, which belong to the *Trichodermaviridescens* complex (Jaklitsch et al. 2013), may still be identified as *T.viridescens*. *T.paraviridescens*, *T.trixiae* and *T.appalachiense* may be identified as the same *Trichoderma* species.

*Trichoderma* species cannot be identified by phylogenetic analysis without considering the sequence similarity values. Therefore, Cai and Druzhinina (2021) developed a protocol for molecular identification of *Trichoderma* that requires analysis of the three DNA barcodes (ITS, *tef1*-α and *rpb2*). Molecular identification of *Trichoderma* species can be achieved, based on the analysis of sequence similarities between the query strain and the reference strains that are analysed for *tef1*-α (≥ 97%) and *rpb2* (≥ 99%). If this condition is not met, the query strain may be a new species of *Trichoderma* and the new species hypothesis can be made, based on sequence similarities and phylogenetic concordance, i.e. analysis of single loci tree topologies for *tef1*-α and *rpb2* and must be verified, based on morphology. In the identification process of the new species, we made full reference to this protocol and there were sufficient differences in sequence similarity between the newly-identified species and the reference species, as well as significant differences in morphological characteristics. According to Jaklitsch et al. (2013), the morphology of *T.viridialbum*, *T.viridarium* and *T.sempervirentis* (meeting the *sp*∃!(*rpb2*_99_≅*tef1*_97_) standard) shows a high degree of similarity and should still be identified as *T.viridescens*. This also fully verified that the identification protocol developed by Cai and Druzhinina (2021) is helpful to ensure the accuracy of *Trichoderma* species identification, which is worth promoting and applying, especially for the identification of *Trichoderma* species.

## Supplementary Material

XML Treatment for Trichoderma hailarense

XML Treatment for Trichoderma macrofasciculatum

XML Treatment for Trichoderma nordicum

XML Treatment for Trichoderma shangrilaense

XML Treatment for Trichoderma vadicola

## Supplementary materials

Supplementary material 1 Five new species of *Trichoderma* from moist soils in China Data type COL Explanation note *Trichodermahailarense* G.Z. Zhang, sp. nov.; *Trichodermamacrofasciculatum* G.Z. Zhang, sp. nov.; *Trichodermanordicum* G.Z. Zhang, sp. nov.; *Trichodermashangrilaense* G.Z. Zhang, sp. nov.; *Trichodermavadicola* G.Z. Zhang, sp. nov. https://binary.pensoft.net/file/647561This dataset is made available under the Open Database License (http://opendatacommons.org/licenses/odbl/1.0/). The Open Database License (ODbL) is a license agreement intended to allow users to freely share, modify, and use this Dataset while maintaining this same freedom for others, provided that the original source and author(s) are credited.Guang-Zhi Zhang, He-Tong Yang, Xin-Jian Zhang, Fang-Yuan Zhou, Xiao-Qing Wu, Xue-Ying Xie, Xiao-Yan Zhao, Hong-Zi Zhou
