# Supplementary material for: ﻿Five new species of Trichoderma from moist soils in China
Source: MycoKeys. 2022 Feb 17;87:133–57. doi: 10.3897/mycokeys.87.76085 (PMC8873192; doi:10.3897/mycokeys.87.76085)
Supplement: Supplementary material 1 — Five new species of Trichoderma from moist soils in China [file mycokeys-87-133-s001.doc]

***Trichoderma hailarense* G.Z. Zhang, sp. nov.**

*MycoBank*: MB 821318

*Etymology*: “hailarense” originally found from the Hailer River basin in the Inner Mongolia of China.

*Typification*: CHINA. Inner Mongolia, Hailar, 618 m, isolated from soil, 17 Sep 2016, G.Z. *Zhang* (Holotype WT 17901). Ex-type culture ACCC 39711. *tef1-α* = MH287505, *rpb2* = MH287506.

*Teleomorph*: Unknown.

Growth optimal at 30 ℃, slow at 35 ℃ on all media. Colony radius after 72 h at 30 ℃ 53–56 mm on PDA, 54–56 mm on CMD, 33–37 mm on MEA, and 33–36 mm on SNA. Colony radius after 72 h at 35 ℃ 13–15 mm on PDA, 10–14 mm on CMD, 9–12 mm on MEA, and 10–12 mm on SNA. Aerial mycelia abundant, arachnoid on PDA after 72 h at 25 ℃ under 12 h photoperiod. Conidiation started around the inoculation point after 7 days on PDA, with relatively few or small conidia. Diffusing pigment or distinctive odor absent. Conidiation started around the inoculation point after 7 days on MEA, forming a few large pustules, cream yellow. On SNA, aerial mycelia were few, forming a few large pustules around the inoculation point in age, cream yellow. Conidiophores and branches narrow and flexuous, tending to be regularly verticillate, forming a pyramidal structure, with each branch terminating in a cruciate whorl of up to 5 phialides. Phialides, lageniform, (8.0–)9.4–13.1(–15.5)×(2.5–)3.0–3.5(–3.6) μm (mean 11.2×3.3 μm), base 1.8–2.5 μm (mean 2.1 μm); phialide length/width ratio (2.33–)2.7 –4.4(–5.9) (mean 3.4). Conidia, obovoid, (4.2–)4.3–4.7(–4.9)×(3.4–)3.6–3.9(–4.1) μm (mean 4.5×3.7 μm), length/width ratio 1.1–1.4 (mean1.2), delicately roughened. Chlamydospores: (7.0–) 7.5–8.2(–8.5)×(6.5–)7.0–7.5(–8.3) μm.

Distribution: China. Inner Mongolia.

*Additional specimen examined*: CHINA. Inner Mongolia, [Hulun](javascript:;) [Buir](javascript:;), 610 m, isolated from soil , 17 Sep 2016, *J.D. Hu* (WT17905).

*Notes*: Phylogenetically *Trichoderma hailarense* is related to *T. gamsii* and *T. neokoningii* (Fig. 1), the sequence similarity of *rpb2* with *T. gamsii* S488 was 97.32%, sequence similarity of *tef1-α* with *T. gamsii* S488 was 97.43% and 96.66%. Estimate the pairwise similarity of *rpb2* with the representative sequences of *T. gamsii* (JN133561) (Bissett et al. 2015), similarity value was 97.36%, sequence similarity of *tef1-α* (DQ307541; Bissett et al. 2015) was 94.92%. The sequence similarity of *rpb2* with *T. neokoningii* CBS120070 was 96.86%, sequence similarity of *tef1-α* with *T. neokoningii* CBS120070 was 96.66%. That does not meet the *sp***∃**!(*rpb2*_99_≅*tef1*_97_) standard for *T. gamsii* and *T. neokoningii*. Morphologically, colonies of *T. gamsii* and *T. neokoningii* on PDA formed conidia sporadically or in hemispherical pustules, and conidia of *T. gamsii* and *T. neokoningii* were ellipsoidal to oblong, smooth-walled (Jaklitsch et al. 2006). However, colonies of *T. hailarense* did not form conidia on PDA, and conidia of *T. hailarense* on other media were obovoid, delicately roughened, and easily distinguished from those of *T. gamsii* and *T. neokoningii*.

***Trichoderma macrofasciculatum* G.Z. Zhang, sp. nov.**

*MycoBank*: MB821299

*Etymology*: “macrofasciculatum” describes the morphological feature of the conidiation, conidiophores aggregated into large fascicles in concentric rings, white.

*Typification*: CHINA, Sichuan, Nine-Village Valley, 2405 m, isolated from soil, 24 Sep 2016, G.Z. *Zhang* (Holotype WT 37805), Ex-type culture ACCC 39712. *tef1-α* = MH287509, *rpb2* = MH287493.

*Teleomorph*: Unknown.

Growth optimum at 20 ℃, slow or limited at 30 ℃, absent at 35 ℃. Colony radius after 72 h at 25 ℃ 21–24 mm on PDA, 23–27 mm on CMD, 17–20 mm on MEA, and 12–16 mm on SNA.

Aerial mycelia abundant on PDA and MEA after incubation for 72 h at 25 ℃ under 12 h photoperiod. Conidiation typically in pustules in concentric rings on PDA, solitary or aggregated, producing a farinose to granular mat. Diameter of pustules up to 2.2 mm, pompon-like, white. Diffusing pigment and distinct odor absent. Conidiation on MEA typically in pustules in concentric rings, pompon-like as on PDA. On CMD, aerial mycelia sparsely developed. Conidiation aggregated in sporadic pustules near the colony margin, white. On SNA, aerial mycelia few, and conidiation not observed. Conidiophores and branches irregularly branched in a dendriform structure, with each branch terminating in a cruciate whorl of up to five phialides. Hyphal septa clearly visible. Phialides, flask-shaped, often curved, (4.9–)5.6–7.8(–8.8)×(2.8–)3.0–3.2(–3.4) μm (mean 6.7×3.1 μm), 1.8–2.6 μm (mean 2.2 μm) near base; phialide length/width ratio (1.5–)1.8–2.4(–2.8) (mean 2.1). Conidia, subglobose to ellipsoid, hyaline, smooth, with one or few distinctly verrucose, (2.6–)2.8–3.3(–3.6)×(2.4–)2.5–2.7(–2.9) μm (mean 3.0×2.6 μm), length/width ratio 1.0–1.3 (mean 1.2), Chlamydospores not observed.

*Distribution*: China, Sichuan province.

*Additional material examined*: CHINA, Sichuan, Nine-Village Valley, 2405 m, isolated from soil, 24 Sep 2016, G.Z. *Zhang* (WT 37810).

*Notes*: Phylogenetically *Trichoderma macrofasciculatum* WT 37805 is related to *T. polysporum* represented by C.P.K. 3131 in the “Polysporum” clade (Fig. 1), but the sequence similarities of *rpb2* and *tef1-α* between these species were only 96.41% and 92.81%, with 94 and 41 bp differences among 1311 and 1152 bp. *Trichoderma macrofasciculatum* cannot grow at 35 ℃ as *T. polysporum*, and the former formes large and white pustules in concentric rings at 25 ℃, elongations were rarely observed and conidia had few guttules, which are distinct from *T. polysporum* (Lu et al. 2004).

***Trichoderma*** ***nordicum* G.Z. Zhang, sp. nov.**

*MycoBank*: MB8212301

*Etymology*: “nord” means found in the nord of China.

*Holotype*: CHINA, Beijing, Yu-yuan-tan Park, 43 m, isolated from soil, 27 Oct 2016, G.Z. Zhang (Holotype WT 13001), Ex-type culture ACCC 39713. *tef1-α* = MH287503, *rpb2* = MH287504.

*Teleomorph*: Unknown.

Growth optimal at 25 ℃, slow or limited at 30 ℃, absent at 35 ℃. Colonies grew fast on PDA, CMD, and MEA and slow on SNA. Colony radius after 72 h at 25 ℃ 67–71 mm on PDA, 68–71 mm on CMD, 51–55 mm on MEA, and 21–24 mm on SNA. Aerial mycelia sparse on PDA after 72 h at 25 ℃ under 12 h photoperiod, and conidiation developed within 48 h beginning at the inoculation point and progressed around, grey white at first and slowly turning green. Diffusing pigment or distinctive odor absent.Aerial mycelia sparse and [flocculence](javascript:void(0);) on MEA after 72 h at 20 ℃ under 12 h photoperiod. Conidia developed within 48 h beginning near the colony margin on MEA, grey white at first and slowly turning green, transparent liquid secreted. Aerial mycelia few On SNA and CMD after 72 h at 25 ℃, conidia formed around the inoculation point and in distinct concentric rings after 96 h under 12 h photoperiod on SNA and CMD, diffusing pigment not produced.

Conidiophores and branches narrow and flexuous, tending to be regularly verticillate forming a pyramidal structure, each branch terminating in a cruciate whorl of up to five phialides. Phialides, lageniform, (6.2–)7.2–10.3(–12.9)×(2.6–)2.9–3.2(–3.4) μm (mean 8.8×3.1 μm), 1.6–2.3 μm (mean 1.9 μm) near base; phialide length/width ratio (2.1–)2.4 –3.4(–4.3) (mean 2.9). On PDA, phialides curved, distinguished from those on other media. Conidia, globose to obovoidal, (4.1–) 4.4–4.8(–5.0)×(4.0–)4.1–4.4(–4.6) μm (mean 4.6×4.3 μm), length/width ratio 1.0–1.2 (mean1.1). Chlamydospores sometimes present, (8.7–)9.8×10.4(–12.5) μm.

*Distribution*: China, Beijing and Hebei.

*Additional specimen* *examined*: China. Hebei, Bai-yang lake, 19 m, isolated from soil, 15 Sep 2016, *J.S. Li* (WT 61001).

*Notes*: Phylogenetically *T.* *nordicum* is related to *T.* *atroviride* (Fig. 1), but the sequence similarities of *rpb2* and *tef1-α* were 97.91% and 95.58%, respectively. That does not meet the *sp***∃**!(*rpb2*_99_≅*tef1*_97_) standard for *T. atroviride* or other known *Trichoderma* species. Morphologically, conidiophores of *T.* *atroviride* typically unilateral, conidia subglobose to ovoidal. Conidiophores of *T. nordicum* were more complexly branched; conidia globose to obovoidal, larger than those of *T.* *atroviride*; transparent liquid secreted on MEA, easily distinguished from that of *T.* *atroviride* (Dodd et al. 2003).

***Trichoderma shangrilaense* G.Z. Zhang, sp. nov.**

*MycoBank*: MB 821300

*Etymology*: “shangrilaense” was originally found at Shangrila in Yunnan Province of China.

*Typification*: China. Yunnan, Pudacuo National Park, 3611 m, isolated from soil, 21 Jun 2016, G.Z. Zhang (Holotype WT 34004), Ex-type culture ACCC 39714. *tef1-α* = MH287495, *rpb2* = MH287496.

*Teleomorph*: Unknown.

Growth optimal at 20 ℃, slow and limited at 25 ℃, and absent at 30 ℃ or 35 ℃. Colony radius after 72 h at 20 ℃ 19–21 mm on PDA, 23–24 mm on CMD, 19–21 mm on MEA, and 8–11 mm on SNA.

Aerial mycelia were abundant, compact on PDA after 7 days at 20 ℃ under 12 h photoperiod, conidiation not easy formed, and a yellow diffusing pigment developed near the inoculation point; after 14 days, conidiation formed pustules that were [unequal](javascript:void(0);) [in](javascript:void(0);) [size](javascript:void(0);) and white. Conidiophores and branches narrow and flexuous, forming a dendriform structure, and irregularly branched, not rebranched, main axis to 4.3–5.0 µm wide, fertile to apex. Phialides, flask-shaped, often curved, (4.5–)5.7–9.0(–11.1)×(2.9–)3.2–3.5(–4.1) μm (mean 7.4×3.4 μm), 1.6–3.4 μm (mean 2.6 μm) near base; phialide length/width ratio (1.5–)2.0 –2.6(–3.0) (mean 2.3). Conidia, obovoid to ellipsoil, smooth, (3.3–)3.5–4.0(–4.4)×(2.8–)3.0–3.3(–3.5) μm (mean 3.8×3.19 μm), length/width ratio 1.1–1.4 (mean 1.2). Chlamydospores not observed.

Colony radius 28–33 mm, aerial mycelia abundant and floccose after 7 days at 20 ℃ under 12 h photoperiod. Conidiation slow to develop on MEA. After about 14 days, pompon-like, white fascicles developed. No diffusing pigment observed. On CMD after 7 days at 20 ℃ under 12 h photoperiod, colony radius 28–33 mm, aerial mycelia few. Conidiation formed flat or cushion-shaped pustules near the colony margin after 21 days, and a yellow diffusing pigment developed near the inoculation point. On SNA after 7 days at 20 ℃ under 12 h photoperiod, colony mycelia sparse, and no conidiation formed. After 10 days, pustules scattered around the periphery of the colony. Diffusing pigment not developed.

*Distribution*: China. Yunnan and Sichuan.

*Additional specimen examined*: CHINA. Sichuan, Huanglong nature reserve, 3561 m, isolated from soil, 25 Sep 2016, *Z. Li* (WT 34012).

*Notes*: *Trichoderma shangrilaense* is related to *T. parapiluliferum* (CBS 120921) (Fig. 1), but the sequence similarity of *rpb2* between these two species is 98.93%, and the sequence similarity of *tef1-α* was 96.35%. The sequence similarity of *tef1-α* with the ex-type culture G.J.S. 91-60 (GenBank Accession No. AY937444) was only 92%. Optimum temperature for growth of *T. shangrilaense* was 20 ℃, no growth occurred at 30 ℃ as in *T. parapiluliferum*, and conidiation structures consist of flat or cushion-shaped pustules formed near the colony margin on MEA, SNA, and CMD. *Trichoderma parapiluliferum*, conidiophore main axis with conspicuous spiral sterile apical elongations, conidia ellipsoidal to oblong (Lu et al. 2004). *Trichoderma shangrilaense*, conidiophore main axis fertile to apex, conidia obovoid to ellipsoid, easily distinguished from that of *T. parapiluliferum*.

***Trichoderma vadicola* G.Z. Zhang, sp. nov.**

*MycoBank*: MB 821316

*Etymology*: “vadicola,” from the Latin word, reflects the ecological environment.

*Typification*: China. Shandong, 2 m, isolated from soil, 13 Aug 2016, G.Z. Zhang (Holotype WT 10708), Ex-type culture ACCC 39716. *tef1-α* = MH287499, *rpb2* = MH287511.

*Teleomorph*: Unknown.

Growth optimal at 25 ℃, absent at 35 ℃ on all media. Colony radius after 72 h at 25 ℃ 25–29 mm on PDA, 24–27 mm on CMD, 23–26 mm on MEA, and 22–26 mm on SNA.

Aerial mycelia abundant on PDA after 72 h at 25 ℃ under 12 h photoperiod, forming strands and floccose mat. Conidiation not formed or relatively few. No diffusing pigment or distinctive odor was produced. On MEA after 72 h at 25 ℃ under 12 h photoperiod, aerial mycelia abundant, floccose. After 7 days, mycelia covered the plate, and conidia appeared, effuse, granuliform. On CMD after 72 h at 25 ℃ under 12 h photoperiod, aerial mycelia not observed. After 7 days, mycelia covered the plate, and conidia developing near the colony margin. On SNA after 72 h at 25 ℃ under 12 h photoperiod, aerial mycelia not observed. After 7 days, mycelia covering the plate, aerial mycelia floccose, and conidia formed, effuse.

Conidiophores and branches tending to be regularly verticillate formed a pyramidal structure, each branch terminating in a cruciate whorl of 3–5 phialides. Phialides, lageniform, (8.3–)9.9– 12.3(–15.1) × (2.0–)2.6–3.2(–3.4) μm (mean 11.1×2.9 μm), 1.1–2.9 μm (mean 1.9 μm) near base; phialide length/width ratio (2.7–)3.2–4.6(–6.6) (mean 3.9). Conidia, subglobose or obovoidal, (3.5–)3.7–4.3(–4.8) × (3.2–)3.4– 3.6(–3.8) μm (mean 4.0×3.5 μm), length/width ratio 1.0–1.3 (mean 1.1). Chlamydospores rare.

*Distribution*: CHINA. Shandong and Yunnan.

*Additional specimen examined*: CHINA. Yunnan, Shangri-La, Pudacuo National Park, 3551 m, isolated from soil, 21 Sep 2016, H.T. Yang (WT 10713)

*Notes*: Phylogenetically, *T**richoderma vadicola* is related to *T. bifurcatum* and *T.tardum* (Fig. 1), but the sequence similarity of*rpb2* between these species was 95.60% and 95.42%, respectively, and sequence similarity of *tef1-α* between these species was 95.9% and 96.44%. *T. vadicola* does not meet the *sp***∃**!(*rpb2*_99_≅*tef1*_97_) standard for *T. bifurcatum* and *T. tardum*. Morphologically, colonies of *T. vadicola* and *T. bifurcatum* have similar features, such as abundant aerial hyphae, forming strands and a whitish hairy or floccose mat, conidiation not formed or relatively few. However, conidiophores and branches of *T. vadicola* tending to be regularly verticillate and formed a pyramidal structure, easily distinguished from that of *T. bifurcatum* (Chen and Zhuang 2017a). Aerial hyphae of *T. tardum* inconspicuous on CMD, conidiation not noted within 30 d, conidiophores simple, acremonium-like to verticillium-like, unbranched with solitary phialides or with few simple branches inclined upwards. But Conidiophores of *T. vadicola* regularly verticillate and repeatedly branched toward the base, forming a pyramidal structure, easily distinguished from *T. tardum* (Chen and Zhuang 2017a).
